# Mechanistic studies of gene delivery into mammalian cells by electrical short-circuiting via an aqueous droplet in dielectric oil

**DOI:** 10.1371/journal.pone.0243361

**Published:** 2020-12-04

**Authors:** Hirofumi Kurita, Hirohito Nihonyanagi, Yuki Watanabe, Kenta Sugano, Ryuto Shinozaki, Kenta Kishikawa, Rika Numano, Kazunori Takashima

**Affiliations:** Department of Applied Chemistry and Life Science, Toyohashi University of Technology, Toyohashi, Aichi, Japan; Xiangtan University, CHINA

## Abstract

We have developed a novel methodology for the delivery of cell-impermeable molecules, based on electrical short-circuiting via a water droplet in dielectric oil. When a cell suspension droplet is placed between a pair of electrodes with an intense DC electric field, droplet bouncing and droplet deformation, which results in an instantaneous short-circuit, can be induced, depending on the electric field strength. We have demonstrated successful transfection of various mammalian cells using the short-circuiting; however, the molecular mechanism remains to be elucidated. In this study, flow cytometric assays were performed with Jurkat cells. An aqueous droplet containing Jurkat cells and plasmids carrying fluorescent proteins was treated with droplet bouncing or short-circuiting. The short-circuiting resulted in sufficient cell viability and fluorescent protein expression after 24 hours’ incubation. In contrast, droplet bouncing did not result in successful gene transfection. Transient membrane pore formation was investigated by uptake of a cell-impermeable fluorescence dye YO-PRO-1 and the influx of calcium ions. As a result, short-circuiting increased YO-PRO-1 fluorescence intensity and intracellular calcium ion concentration, but droplet bouncing did not. We also investigated the contribution of endocytosis to the transfection. The pre-treatment of cells with endocytosis inhibitors decreased the efficiency of gene transfection in a concentration-dependent manner. Besides, the use of pH-sensitive dye conjugates indicated the formation of an acidic environment in the endosomes after the short-circuiting. Endocytosis is a possible mechanism for the intracellular delivery of exogenous DNA.

## Introduction

Delivery of cell-impermeable molecules, such as DNA, RNA, proteins, antibodies, and drugs, is essential for basic research in the life sciences and medicine [1]. For example, the establishment of induced pluripotent stem (iPS) cells requires the intracellular delivery of four specific transcriptional factors [2, 3]. Plant genetic engineering is the most common approach to improving plant yields, nutrition, and pest resistance [4]. Genome editing, which also requires gene transfection or protein introduction, has been widely used in current life science research [5–9]. In most applications, delivery methods need to provide both high delivery efficiency and high cell viability. Therefore, novel techniques for delivering extracellular molecules into cells are needed.

Among the various methods previously developed and commercialized, electroporation (EP) is the most well-established physical approach [10, 11]. In EP, the application of an external electric field to cells induces transient membrane pore formation and the incorporation of molecules which could not normally permeate into cells. For suspended cells, a cell solution is supplied in an electroporation cuvette connected to electroporators, which produce short electrical pulses at a high voltage. For adhesive cells or tissues, electroporation needles are used. Many investigations have been conducted into the mechanisms underlying gene electrotransfer [12–19]. These reports suggest that the electrotransfection process includes not only transient membrane pore formation but also endocytosis and endosomal trafficking of DNA. Improvement of transfection efficiency has been investigated using mechanistic analyses [19, 20]. Recently, various types of advanced EP devices have been developed [21–24]. Application of microfabrication techniques and microfluidics to the development of EP devices has been investigated [24–26]. The application of electrostatic actuation of water droplets in dielectric oil has also been investigated. Im et al. reported electrophoretic actuation of water-in-oil droplets containing living cells [27], and also successfully investigated gene transfection of microalgae [28–30] and Jurkat cells [31] using their droplet-based system.

We have developed a novel electroporation method based on electrical short-circuiting via a water droplet in dielectric oil; this methodology is termed “droplet EP” in this paper. When a cell suspension droplet is placed between a pair of electrodes with an intense DC electric field, droplet bouncing and droplet deformation can be induced, depending on the strength of the electric field. Droplet deformation results in an instantaneous short-circuit caused by the droplet elongating and bridging two electrodes simultaneously. We have demonstrated transformation of *Escherichia coli* [32] using droplet bouncing in a DC electric field and gene transfection into various mammalian cells using the short-circuiting [33]. Recently, successful delivery of exogenous DNA into bovine and swine fibroblasts by droplet EP has also been reported [34]. It is, however, difficult to transfect plasmids into bovine fibroblasts using conventional lipofection methods. Previous investigations have shown that droplet EP can be used for the delivery of proteins into animal sperm. Droplet EP can be tailored by varying the following experimental parameters: applied voltage, number of short-circuits, type of medium (electrical conductivity), concentration of exogenous DNA, and size of the droplet [35]. In the previous investigation, the relative number of viable cells and luciferase activity was measured simultaneously in a cell culture population, using a luciferase-expressing plasmid DNA and a 96-well assay system. Although this approach is very useful for determining the way in which experimental parameters influence transfection, the data obtained from a microplate reader are the sum of the amount of luminescence in the well. Measurements from both living and dead cells are included, so the sizes of the populations of viable cells and transfected cells cannot be obtained. The process of transferring droplets from the oil to a 96-well plate may affect the number of viable cells, because it is difficult to recover all of the treated cells.

In this paper, we performed flow cytometric assays to obtain more precise data and investigate the transfection process. Aqueous droplets containing mammalian cells and plasmids carrying fluorescent proteins were treated with droplet bouncing or short-circuiting, and then cell viability and transfection efficiency were evaluated 24 hours after treatment, using dead cell staining fluorescent dyes. Although our previous investigation using a 96-well assay system showed that short-circuiting is critical for gene electrotransfer [35], we confirmed the difference between droplet bouncing and short-circuiting on gene electrotransfer. Then, the gene electrotransfer mechanism was investigated by the flow cytometric assays. In this paper, transient membrane pore formation and endocytosis stimulated by short-circuiting were elucidated. The preliminary investigations have already shown that short-circuiting stimulates transient membrane pore formation [33, 35]; however, the difference between droplet bouncing and short-circuiting on transient membrane pore formation was not elucidated. The uptake of a live cell-impermeable nucleic acid staining dye and the influx of calcium ions (Ca^2+^) were monitored by flow cytometry. Furthermore, endocytosis contributing to gene transfection was investigated using cells treated with endocytosis inhibitors before short-circuiting. The formation of endocytic vesicles stimulated by short-circuiting was also monitored. The cells were incubated with pH-sensitive fluorescent dextran conjugates after treatment, and the increase in fluorescence of the cells, indicating a pH decrease in endocytic vesicles, was measured.

## Materials and methods

### Cell culture and plasmid DNA preparation

Jurkat cells, an immortalized line of human acute T cell lymphocyte cells, were grown in RPMI-1640 with l-glutamine and phenol red (FUJIFILM Wako Pure Chemical), 10% fetal bovine serum (FBS, One Shot fetal bovine serum, Thermo Fisher Scientific), 100 units/ml penicillin, and 100 *μ*g/ml streptomycin (PS, FUJIFILM Wako Pure Chemical) at 37°C, 5% CO_2_. Prior to use cells were harvested by centrifugation and resuspended in RPMI-1640 without FBS, and the cell concentration was adjusted.

Plasmid DNA expressing Venus yellow fluorescent protein (YFP) was provided by Prof. A. Miyawaki at RIKEN [36]. The plasmid DNA was used for the fluorescent protein expression and flow cytometry assays. The amplification and purification procedures were as described in a previous paper [35]. Following purification, endotoxin was removed using an endotoxin removal kit (MiraCLEAN, Mirus). The purified plasmid DNA was dissolved in sterile water, and the DNA concentration determined using a UV-vis spectrometer (GeneQuant1300, GE Healthcare).

### Experimental setup

**Fig 1:**
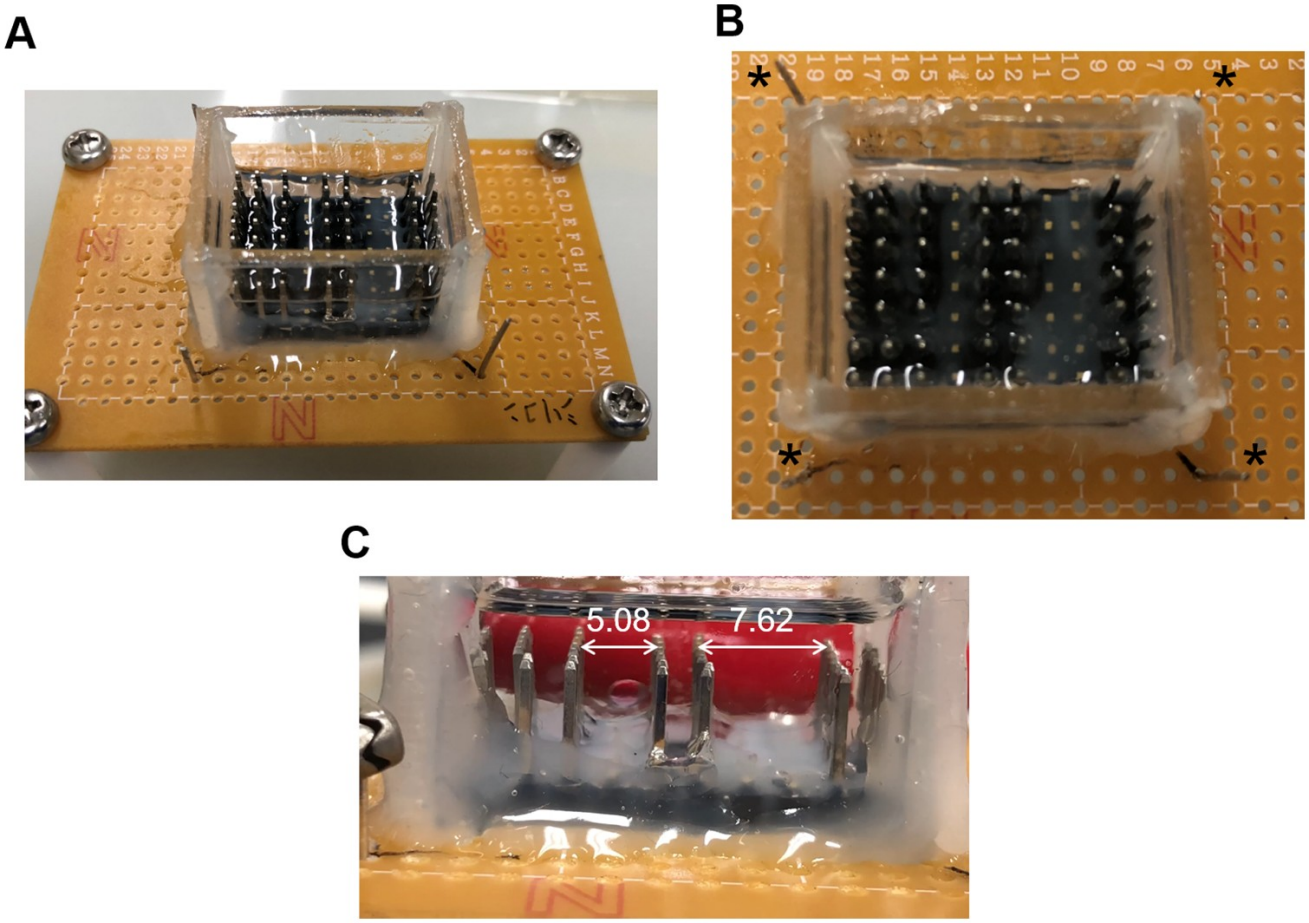
Experimental setup for electrostatic actuation of a water droplet in dielectric oil. Overview (A), top view (B), and side view (C) of the experimental apparatus. * in B indicates connection to a DC HV power supply. The numerical value in C indicates the gap between the electrodes, in millimeters.

Fig 1 shows the experimental apparatus for actuation of a water droplet in dielectric oil. The apparatus was fabricated as described in previous papers [28, 30, 31]. Pin headers (2 × 10 pins with 2.54 mm pitch, RS components) were assembled on a printed circuit board (Sunhayato) by soldering. The array of electrodes was enclosed with a plastic plate (1.7 mm thickness, Tamiya) to contain the dielectric oil. To prevent oil leakage, the bottom of the oil reservoir was coated with polydimethylsiloxane (PDMS). The electrodes of the base were coated with silicone glue to prevent unauthorized discharge. The gap between the electrodes was set to 5.08 or 7.62 mm by cutting the pin electrodes. Jumper wires were used for the connection between the basement and a DC high voltage (HV) power supply (HAR-30R10, Matsusada Precision Inc.).

### Actuation of a water droplet in dielectric oil

The apparatus was filled first with 1.5 ml fluorocarbon oil (Fluorinert FC-43, 3M), and then 1 ml silicone oil (KF96-100CS, 100 cSt kinematic viscosity, 2.74 dielectric constant, 965 kg/m^3^ density, Shin-Etsu Chemical Co.) without surfactant was added. The cell suspension (3.0 *μ*l) was dispensed in the silicone oil and a high voltage was supplied using the DC HV power supply.

Two modes of droplet actuation were used in this study: droplet bouncing, and short-circuiting via the droplet. For droplet bouncing, 1.5 kV/5.08 mm (2.95 kV/cm) or 5.0 kV/7.62 mm (6.56 kV/cm) was applied to the electrode. A high voltage was applied for 10 minutes to continue the droplet bouncing. When the applied voltage was set to 2.5 kV or higher, with a 5.08 mm gap, the higher electric field can induce droplet elongation, leading to instantaneous short-circuiting caused by the droplet contacting the two electrodes simultaneously. The short-circuiting produced a distinctive sound, and the DC HV power supply was manually turned off immediately. The number of shorts was set to one, except for the experiment which investigated the effect of the number of shorts. To investigate the effect of the number of shorts, this process was repeated a pre-designated number of times. The droplet bouncing and short-circuiting were recorded using a smart phone. Following the DC HV application, the droplet was recovered and transferred to an appropriate medium for further experiments.

### Measurement of transfection efficiency and cell viability

A 3.0 *μ*l aliquot of RPMI-1640 without serum, containing 1.0 × 10^5^ Jurkat cells and 0.2 *μ*g of the Venus YFP-expressing plasmid DNA was added to the silicone oil. After droplet bouncing or short-circuiting, the droplet was recovered into a microcentrifuge tube containing RPMI-1640/FBS/PS, and the cell suspension was transferred to a well in a 24-well cell culture plate. The cell culture was incubated for 24 hours at 37°C, with 5% CO_2_, harvested by centrifugation, and resuspended in Dulbecco’s phosphate-buffered saline without magnesium chloride and calcium chloride (D-PBS (-), FUJIFILM Wako Pure Chemical). Following resuspension, 7-amino-actinomycin D (7-AAD, Beckman Coulter, Inc.) was added to the cell suspension to stain the dead cells, and the cells were incubated for 20 minutes. YFP expression and cell viability were measured with a CytoFLEX flow cytometer (Beckman Coulter, Inc.). At least 10,000 events were recorded for each experimental point. Raw data acquisition was performed using the CytExpert software. Transfection efficiency was calculated by dividing the number of viable, YFP-expressing, cells by the total number of cells. Viability was calculated by dividing the number of 7-AAD negative cells by the total number of cells. The flow cytometer settings were kept constant for each experiment. Kaluza Analysis 2.1 software (Beckman Coulter, Inc.) was used for all data analysis.

### Uptake of cell-impermeable nucleic acid-binding fluorophores

The formation of transient membrane pores induced by short-circuiting was confirmed using a live cell impermeable nucleic acid staining dye, YO-PRO-1 (Thermo Fisher Scientific) [37]. YO-PRO-1 uptake assay was conducted as previously described, with some modifications [33, 35]. A 3.0 *μ*l droplet containing 1.0×10^5^ Jurkat cells suspended in RPMI-1640 with 1 *μ*M YO-PRO-1 was added to the silicone oil, and droplet bouncing or short-circuiting was performed. After the droplet actuation, the droplet was recovered into a microcentrifuge tube containing RPMI-1640 without serum, then incubated for 40 minutes at 37°C. Following incubation, 7-AAD was added to the cell suspension, which was then incubated for 20 minutes. YO-PRO-1 uptake and cell viability were measured using flow cytometry. YO-PRO-1 uptake efficiency was calculated by dividing the number of YO-PRO-1 positive viable cells by the total number of cells.

### Influx of calcium ions through a transient membrane pore

Jurkat cells were incubated with 4.5 *μ*M Fluo-4 AM (Thermo Fisher Scientific) in D-PBS (-) for 60 minutes at 37°C. Following the loading, the cells were harvested by centrifugation and resuspended in HEPES-buffered saline (Lonza) containing 10 mM CaCl_2_. A 3.0 *μ*l droplet containing 1.0×10^5^ Fluo-4-loaded Jurkat cells suspended in HEPES-buffered saline containing 10 mM CaCl_2_ was added to the silicone oil, and droplet bouncing or short-circuiting was performed. Following the droplet actuation, the droplet was recovered into a microcentrifuge tube containing HEPES-buffered saline without CaCl_2_. The fluorescence intensity of the cells was measured using flow cytometry.

### Effect on gene transfection of pre-treatment of cells with endocytosis inhibitors

To evaluate the contribution of endocytosis to the gene transfection, Jurkat cells were treated with endocytosis inhibitors before transfection. In this study, methyl-*β*-cyclodextrin (M*β*CD, FUJIFILM Wako Pure Chemical) and Pitstop 2-100 (Abcam) were used. The endocytosis inhibitors were added to the cell suspension at a pre-determined final concentration, and the cells were incubated for 30 minutes at 37°C, with 5% CO_2_. Following pre-treatment, the cells were harvested by centrifugation, and resuspended in RPMI-1640 without FBS, and the cell concentration was adjusted. Following the addition of the plasmid DNA, the short-circuiting (3.0 kV, 5.08 mm gap, 1 short) was performed on the pre-treated Jurkat cells, and the transfection efficiency and cell viability 24 hours after transfection were measured, as described above.

### Monitoring endocytosis with pH-sensitive fluorescent dextran conjugates

A 3.0 *μ*l aliquot of RPMI-1640 without serum, containing 1.0 × 10^5^ Jurkat cells, was added to the silicone oil. Following droplet bouncing or short-circuiting, the Jurkat cells were incubated in RPMI-1640 with 20 *μ*g/ml pHrodo Green dextran (10,000 MW, for Endocytosis, Thermo Fisher Scientific) for 30 minutes at 37°C. Following incubation, the fluorescence intensity of the cells was measured using flow cytometry.

## Results

### Actuation of water droplets in dielectric oil and behavior of Jurkat cells in droplets

S1 Video shows a droplet bouncing, and S2 Video shows short-circuiting caused by the droplet, recorded from a lateral view. Once an aqueous droplet in dielectric oil under a DC electric field was charged by induction charging, Coulomb forces moved the charged droplet toward the electrode with opposite polarity. When the droplet touched with the electrode, the polarity of the droplet changed, and the droplet moved toward the counter electrode. The droplet bouncing continued during the application of HV DC. From S1 Video, the droplet touched the electrodes five times per second. The electrode gap was 5.08 mm; therefore, the average velocity was calculated as approximately 25 mm/s. When a higher-strength electric field was applied, deformation of the droplets and short-circuiting by the droplet occurred, as shown in S2 Video.

**Fig 2:**
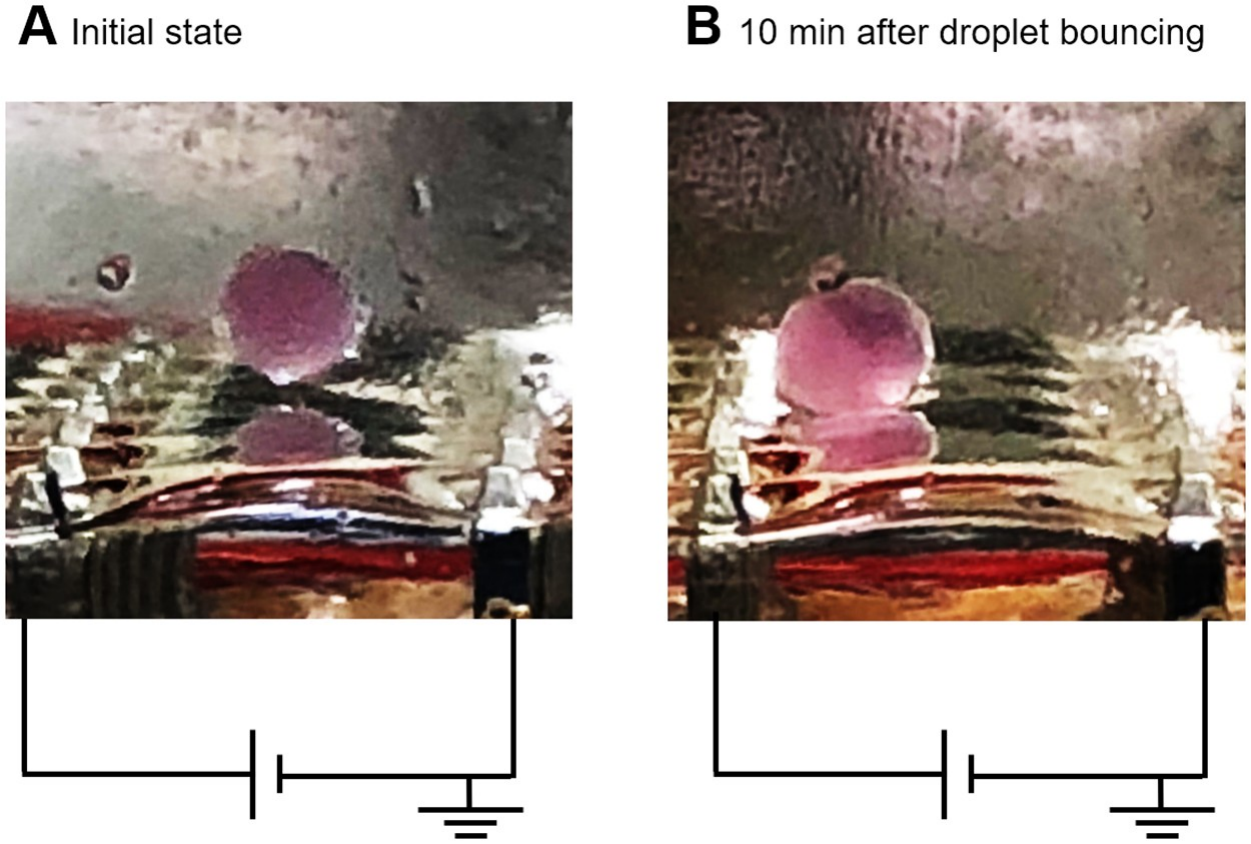
Behavior of Jurkat cells in a droplet during bouncing. A 3.0 *μ*l aliquot containing 1.0 × 10^5^ Jurkat cells in RPMI-1640 without serum was added to the silicone oil. A charge of 1.5 kV was applied to the electrodes, with a 5.08 mm gap, to initiate droplet bouncing. High voltage was applied for 10 minutes to continue the droplet bouncing. A: Initial state of the droplet bouncing. B: After 10 minutes of droplet bouncing.

In our previous work, we used aqueous droplets containing less than 1.0 × 10^4^ cells to perform droplet actuation in 24- or 96-well microplates [33–35]. It was therefore difficult to observe the behavior of the cells during droplet actuation. The experimental apparatus fabricated in this study allowed us to observe the behavior of the droplet and the encapsulated cells simultaneously. Fig 2 shows behavior of Jurkat cells in the droplet during droplet bouncing. At the initial state of the droplet bouncing shown in Fig 2A, the cells in the droplet seemed to be uniformly dispersed. After around 10 minutes of droplet bouncing, the cells were localized around the anode side of the droplet, as shown in Fig 2B. This phenomenon became apparent when the number of the cells in an aqueous droplet was increased. S3 and S4 Videos show behavior of cells in a droplet at bouncing. These movies also indicate that the cells were aggregated around the anode side of the droplet.

### Evaluation of cell viability and transfection efficiency

**Fig 3:**
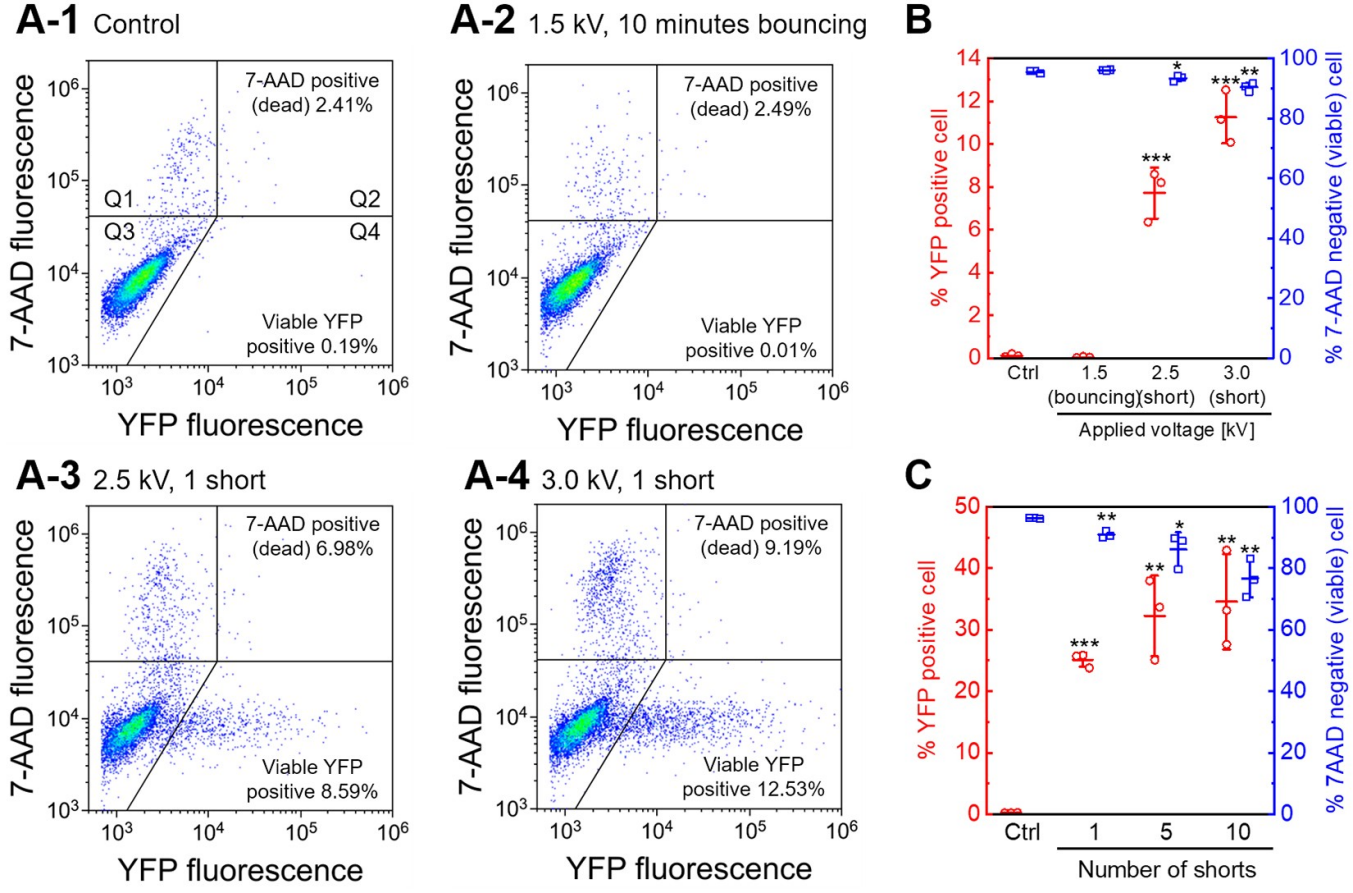
Cell viability and fluorescent protein expression measured using flow cytometry. Jurkat cells were treated with droplet bouncing (1.5 kV, 10 minutes) or short-circuiting via the droplet (2.5 or 3.0 kV, 1 short) and assayed according to 7-AAD uptake for cell death 24 hours after treatment. The electrode gap was 5.08 mm. “Control” indicates a control experiment, in which the droplet containing cells and plasmid DNA was added to the oil and recovered immediately. A: Intensity of fluorescence of YFP and 7-AAD was measured, and plotted using a log scale. The flow cytometry data are displayed as density plots. The plot is divided into four quadrants (Q1, Q2, Q3, and Q4). Quadrants Q1 and Q2 include the 7-AAD positive (dead) cells, Q3 includes YFP negative and 7-AAD negative cells, and Q4 includes YFP positive and 7-AAD negative cells. The percentages of 7-AAD positive (dead) cells (Q1+Q2) and YFP-positive viable cells (Q4) are shown. Results are representative of at least three independent experiments. B: Transfection efficiency and cell viability 24 hours after droplet bouncing or short-circuiting, as determined using flow cytometry. Data are expressed as the mean ± standard deviation (SD) of triplicate measurements. Statistical significance was determined using Student’s *t*-tests, *p < 0.05, **p < 0.01, and ***p < 0.001 vs. ctrl. C: Effect of the number of short-circuits. The voltage was set to 3.0 kV. Data are expressed as the mean ± SD of triplicate measurements. Statistical significance was determined using Student’s *t*-tests, *p < 0.05, **p < 0.01, and ***p < 0.001 vs. ctrl.

**Fig 4:**
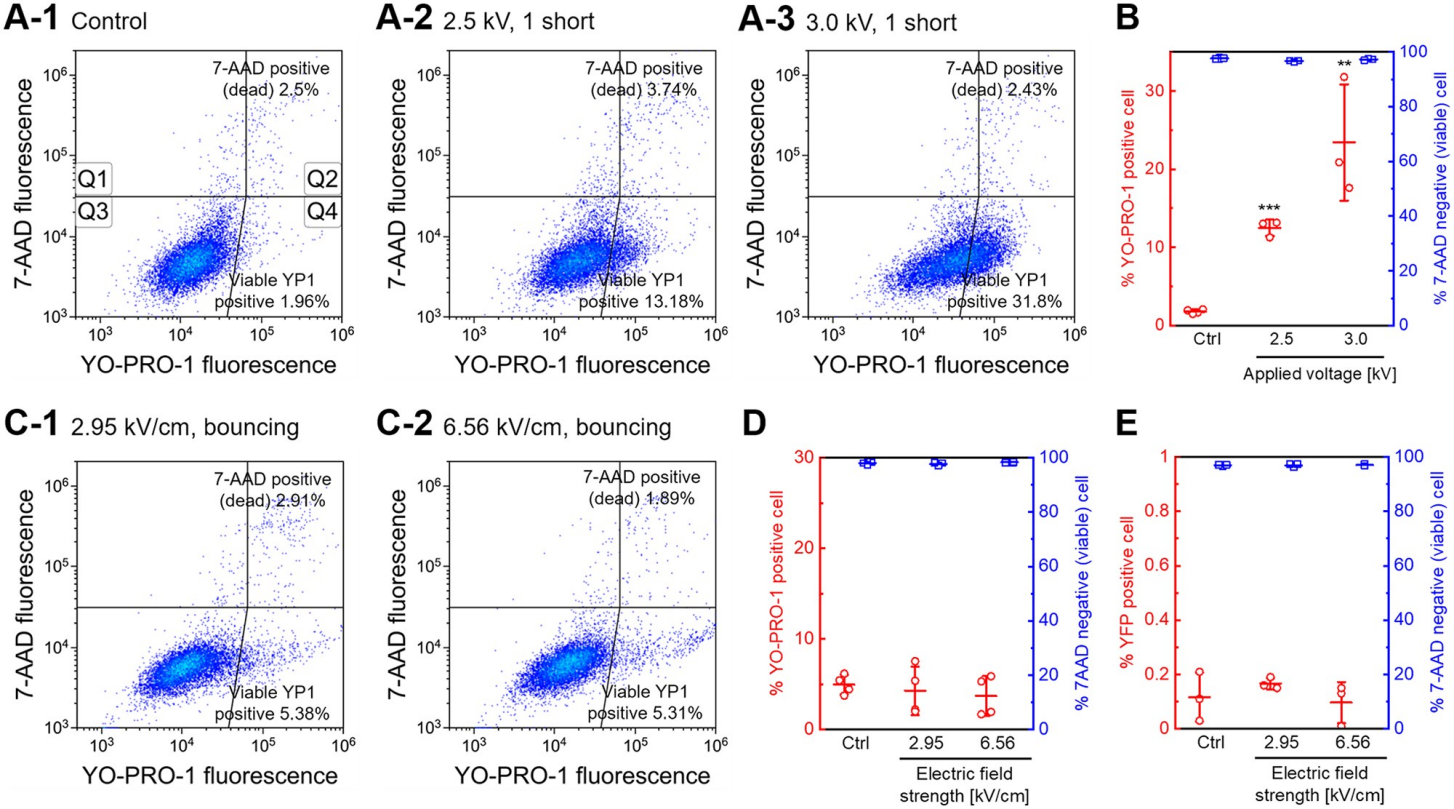
Permeabilization of YO-PRO-1 molecules measured by flow cytometry. A: YO-PRO-1 uptake was induced by short-circuiting via the droplets (2.5 or 3.0 kV, 1 short). The fluorescence intensities were determined for YO-PRO-1 and 7-AAD, and then plotted using a log scale. The flow cytometry data are displayed as density plots. The plot is divided into four quadrants (Q1, Q2, Q3, and Q4). Quadrants Q1 and Q2 contain 7-AAD positive (dead) cells, Q3 contains YO-PRO-1 negative and 7-AAD negative cells; Q4 contains YO-PRO-1 positive and 7-AAD negative cells. The percentages of 7-AAD positive (dead) cells (Q1+Q2) and YO-PRO-1-positive viable cells (Q4) are shown. Results are representative of at least three independent experiments. B: YO-PRO-1 uptake efficiency and cell viability one hour after short-circuiting, as determined using flow cytometry. Data are expressed as the mean ± SD of at least three measurements. Statistical significance was determined using Student’s *t*-tests, **p < 0.01, ***p < 0.001 vs. ctrl. C: YO-PRO-1 uptake was not stimulated by droplet bouncing for 10 minutes. The result is representative of four independent experiments. D: YO-PRO-1 uptake efficiency and cell viability one hour after droplet bouncing. Jurkat cells were treated with droplet bouncing for 10 minutes with the indicated electric field strength. Data are expressed as the mean ± SD of at quadruplicate measurements. E: Gene transfection by droplet bouncing with a more intense electric field strength. Data are expressed as the mean ± SD of triplicate measurements.

Fig 3 shows fluorescent protein expression and cell viability as measured using flow cytometry. Experimental data were obtained using a cell suspension prepared with a single cell culture. The experiments for different sets of parameters, Fig 3B and 3C, were performed using different cell cultures. The health of the cells, number of passages, and concentration of cells before the transfection were therefore not completely identical between experiments. Fig 3A shows typical flow cytometry data, displayed as density plots. As shown in Fig 3A-2, droplet bouncing resulted in no significant changes in the density plots relative to the control shown in Fig 3A-1. These results suggest that gene transfection by droplet bouncing did not succeed under the experimental conditions. However, Fig 3A-3 and 3A-4 clearly show an increase in the population of YFP-expressing, viable, cells relative to control, although the size of the population of dead cells was also increased. Most YFP-expressing cells were viable. Fig 3B shows the cell viability and transfection efficiency 24 hours after droplet bouncing or short-circuiting, as determined from the density plots. Short-circuiting via the droplets resulted in a significant increase in the transfection efficiency relative to the control. A slight decrease in cell viability was observed, but more than 90% of the cells were viable in each condition. Higher transfection efficiency was observed when using 3.0 kV of applied voltage than with 2.5 kV (*p* = 0.023, determined using Student’s *t*-test). Fig 3C shows the effect of the number of short-circuits. Short-circuiting produces a distinctive sound, and the DC HV power supply was turned off immediately. Thus, the number of shorts could be counted. Cell viability decreased with increasing the number of short-circuits, although a slight increase in transfection efficiency was observed. Fig 3B and 3C suggest that differences in the mode of droplet actuation were more important to gene transfection than the number of short-circuits.

### Transient membrane pore formation

Fig 4 shows the results of YO-PRO-1 uptake assays. The live cell-impermeable nucleic acid staining dye YO-PRO-1 was used to investigate transient pore formation on the cell membrane. However, both transient pore formation and the presence of dead cells can lead to an increase in the intensity of YO-PRO-1 fluorescence. Therefore, 7-AAD was added to the sample following incubation after droplet bouncing or short-circuiting. Fig 4A shows typical flow cytometry data displayed as density plots. Short-circuiting resulted in changes in YO-PRO-1 fluorescence intensity relative to the control experiment shown in Fig 4A-1. Fig 4B shows the percentage of YO-PRO-1-positive viable cells and 7-AAD negative (viable) cells one hour after short-circuiting, as determined by flow cytometry. A significant increase in the population of YO-PRO-1 positive viable cells was observed after short-circuiting. Short-circuiting using 3.0 kV of applied voltage resulted in more than 20% in the population of YO-PRO-1 positive viable cells. A higher population of YO-PRO-1 positive viable cells was observed by comparing 2.5 kV and 3.0 kV (*p* = 0.065, determined using Student’s *t*-test). The population of 7-AAD negative cells did not, however, change in each experimental condition. This result suggests that the increase in YO-PRO-1 fluorescence was due to transient membrane pore formation.

Although droplet bouncing did not succeed gene transfection, YO-PRO-1 uptake assays with droplet bouncing were performed. In this investigation, higher strength electric fields were applied for droplet bouncing. The gap between the electrodes was extended to avoid short-circuiting with the higher voltage. Fig 4C shows typical flow cytometry data from the YO-PRO-1 uptake assay. In this experiment, 6.56 kV/cm of DC electric field was applied to continue droplet bouncing for 10 minutes. Each droplet bouncing did not increase in YO-PRO-1 fluorescence intensity, suggesting that transient membrane pore formation was not stimulated by the droplet bouncing. Fig 4D shows the percentage of YO-PRO-1 positive viable cells and 7-AAD negative (viable) cells one hour after droplet bouncing with different electric field strength, as determined by flow cytometry. This figure shows that droplet bouncing did not increase the population of YO-PRO-1 positive cells. Furthermore, the experimental conditions used in Fig 4C and 4D did not succeed gene transfection (Fig 4E).

**Fig 5:**
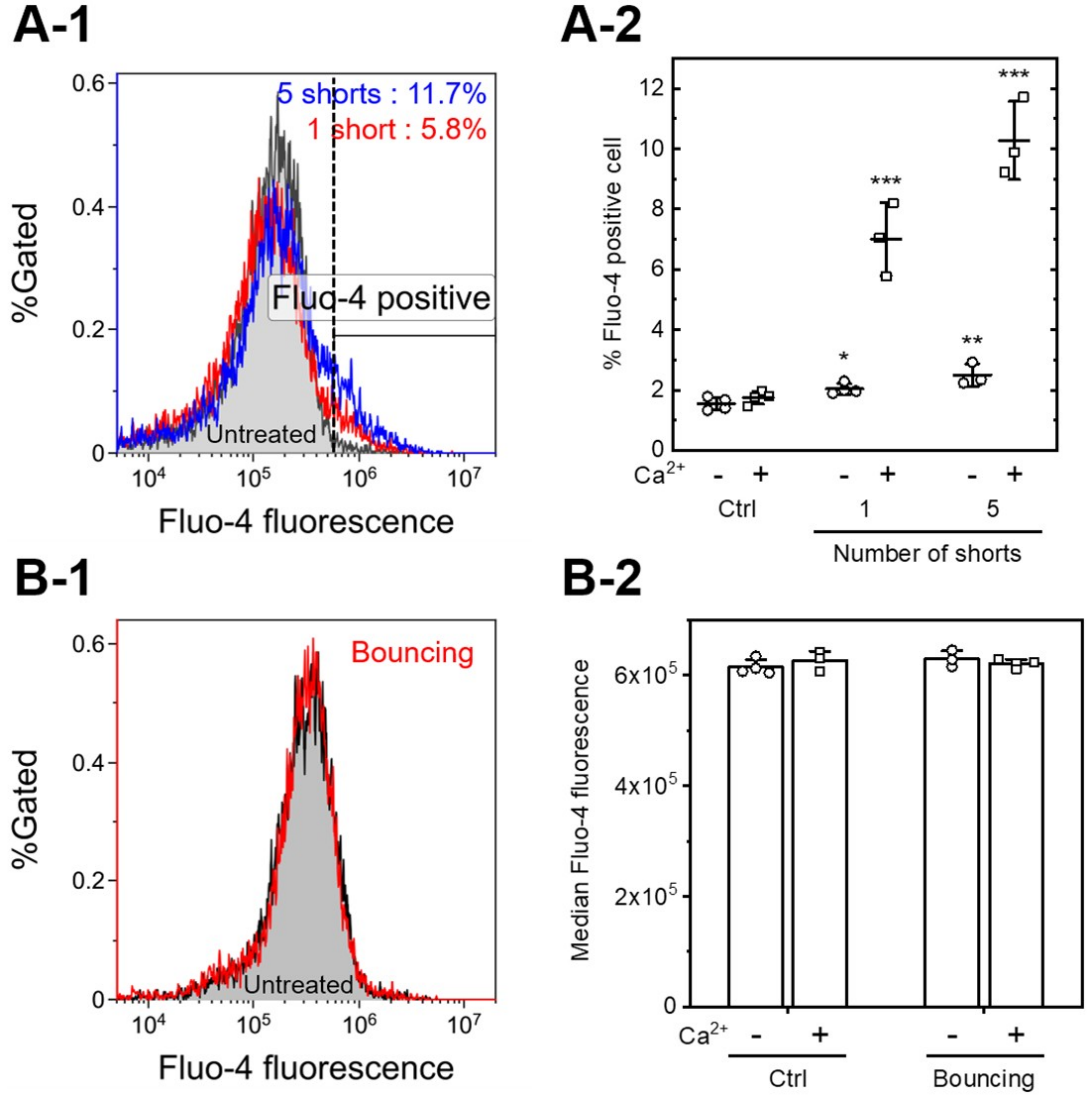
Influx of calcium ions measured by flow cytometry. A: The influx of Ca^2+^ was stimulated by short-circuiting via the droplets (3.0 kV, 1 or 5 shorts). A 3.0 *μ*l droplet containing 1.0×10^5^ Fluo-4-loaded Jurkat cells suspended in HEPES-buffered saline with (+) or without (-) 10 mM CaCl_2_ was added to the silicone oil, and short-circuiting via a droplet was performed. (A-1) Typical flow cytometry histograms. The fluorescence intensity was determined for Fluo-4. The flow cytometry histogram of the untreated control is filled gray. The black dashed line indicates the threshold of the Fluo-4 positive cells. The percentage of the Fluo-4 positive cells is shown. Results are representative of at least three independent experiments. (A-2) The population of Fluo-4 positive cells, as determined using flow cytometry. Data are expressed as the mean ± SD of at least three measurements. Statistical significance was determined using Student’s *t*-tests, *p < 0.05, **p < 0.01, ***p < 0.001 vs. ctrl. B: Droplet bouncing (6.56 kV/cm, 10 minutes) did not increase Fluo-4 fluorescence intensity. (B-1) Typical flow cytometry histograms. A 3.0 *μ*l droplet containing 1.0×10^5^ Fluo-4-loaded Jurkat cells suspended in HEPES-buffered saline with 10 mM CaCl_2_ was added to the silicone oil, and droplet boncing was performed. The flow cytometry histogram of the untreated control is filled gray. The result is representative of four independent experiments. (B-2) Median Fluo-4 fluorescence intensity, as determined using flow cytometry. Data are expressed as the mean ± SD of at least three measurements.

We also investigated the influx of Ca^2+^ to confirm transient membrane pore formation [37]. Fluo-4 was used as an indicator for Ca^2+^ concentrations. The use of the indicator can detect the influx of Ca^2+^ ions into the cell. Fig 5 shows the results of the flow cytometric analysis. Fig 5A-1 shows typical flow cytometry data after short-circuiting displayed as histograms. The applied voltage was set to 3.0 kV, and the number of shorts was set to one or five. Short-circuiting increased in the population of Fluo-4 positive cells relative to the control experiment. Fig 5A-2 shows the percentage of Fluo-4-positive cells after short-circuting, as determined by flow cytometry. A significant increase in the population of Fluo-4 positive cells was observed with short-circuiting. Five shorts increased the population of Fluo-4 positive cells by comparing one short (*p* < 0.05, determined using Student’s *t*-test). Similar experiments were performed with the cell suspension not containing Ca^2+^. Also, short-circuiting using 3.0 kV of applied voltage significantly increased the population of Fluo-4 positive cells. Fig 5B shows the results of the flow cytometric analysis after droplet bouncing. 6.56 kV/cm of DC electric field was applied to continue droplet bouncing for 10 minutes, as same as Fig 4C. The results show that the droplet bouncing did not change in the flow cytometry histograms. The addition of Ca^2+^ did not show the difference in the median Fluo-4 fluorescence intensity by comparing that without Ca^2+^.

### Contribution of endocytosis to gene transfection

**Fig 6:**
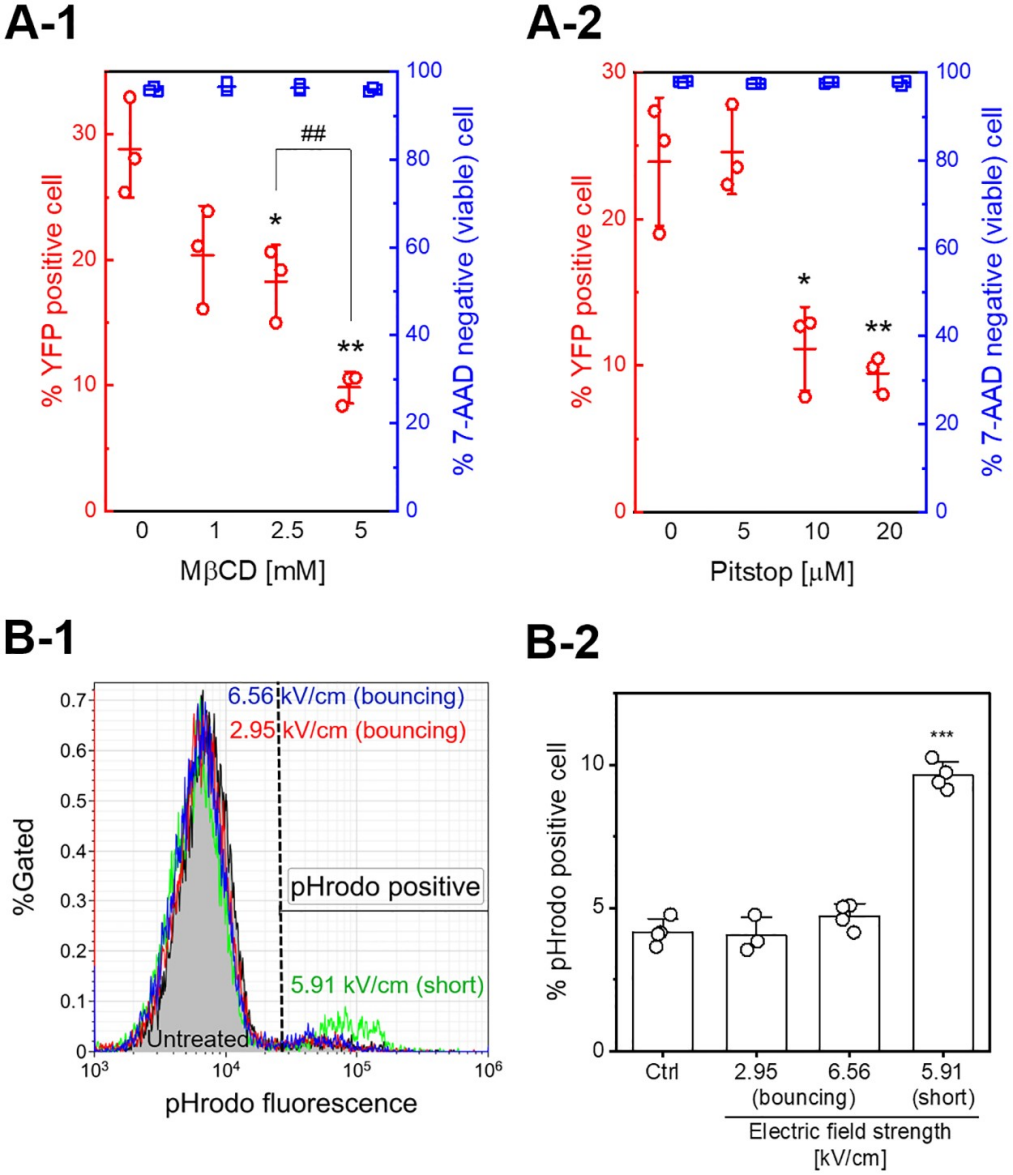
Measurement of endocytosis induced by short-circuiting via an aqueous droplet. A: Effect of pre-treatment of cells with endocytosis inhibitors on gene transfection. Jurkat cells were pre-treated with endocytosis inhibitors, M*β*CD (A-1) and Pitstop 2-100 (A-2), before short-circuiting (3.0 kV, 5.08 mm gap, 1 short) and assayed for cell death using 7-AAD uptake for 24 hours after droplet EP. Data are expressed as the mean ± SD of at three measurements. Statistical significance was determined using Student’s *t*-tests, *p < 0.05 and **p < 0.01 vs. vehicle control, and ##*p* < 0.01. B: Monitoring endocytosis with pH-sensitive fluorescent dextran conjugates. Typical flow cytometry histograms (B-1) and population of pHrodo Green positive cells (B-2) are shown. The black dashed line in B-1 indicates the threshold of the pHrodo Green positive cells. Data are expressed as the mean ± SD of at quadruplicate measurements. Statistical significance was determined using Student’s *t*-tests, ***p < 0.001 vs. untreated control.

Fig 6 shows the results of flow cytometry assays used to investigate the contribution of endocytosis to gene transfection. Fig 6A shows the effect of pre-treatment of the cells with endocytosis inhibitors. Here, M*β*CD, a cholesterol-depleting chemical [38], and Pitstop 2-100, a clathrin inhibitor [39] (IC_50_: 7.5 *μ*M from manufacturer’s datasheet), were used. Flow cytometry assays were performed as in Fig 3. No significant decrease in cell viability 24 hours after gene transfection was observed using either endocytosis inhibitor. Therefore, pre-treatment of cells with endocytosis inhibitors did not affect cell health. Transfection efficiency, however, was significantly decreased. The pre-treatment with M*β*CD decreased transfection efficiency in a concentration-dependent manner (Fig 6A-1). In contrast, Pitstop also resulted in a decrease in transfection efficiency, but no significant decrease was observed by comparing 10 *μ*M and 20 *μ*M (Fig 6A-2).

Fig 6B shows the results of monitoring endocytosis using pH-sensitive fluorescent dextran conjugates and flow cytometry. The fluorescence of the pHrodo Green dye increases as pH decreases from neutral to acidic, so the encapsulation of macromolecules in endocytic vesicles can be monitored. As shown in Fig 6B-1, short-circuiting produced a change in the flow cytometry histograms relative to the untreated control. Fig 6B-2 shows the population of pHrodo Green positive cells. Compared with the untreated control, short-circuiting resulted in a significant increase in the population of pHrodo Green positive cells. These results suggest that endocytosis may contribute to the gene transfection process. Similar experiments were performed with droplet bouncing. The experimental conditions were the same as those used to produce Fig 4C–4E. As shown in Fig 6B, droplet bouncing did not result in increases in the population of pHrodo Green positive cells. This result suggests that droplet bouncing did not stimulate endocytosis.

## Discussion

Flow cytometric assays were performed to investigate the mechanisms underlying novel gene transfection using the application of HV electric fields. In our previous investigations, the cell viability and transfection efficiency of gene transfection was evaluated using fluorescence microscopic images [33], with simultaneous measurement of metabolic activity and luciferase activity in a 96-well assay system [35] involving a small number of cells (less than 10,000 cells). One of the advantages of the droplet-based methodology is that transfection can be performed using small numbers of cells [33]. Therefore, we have not previously performed gene transfection using relatively large numbers of cells. However, since a small number of cells was insufficient for flow cytometry, flow cytometric assays were performed in this study using 1.0 × 10^5^ cells/3 *μ*l droplet. Gene transfection was successfully performed, as shown in Fig 3. This methodology is therefore applicable to a wide range of cell numbers.

By increasing the number of cells in a droplet, it became possible to observe that the cells in the droplet were localized around the anode during droplet bouncing, as shown in Fig 2, S3 and S4 Videos. The cell surface is usually negatively charged; therefore, electrophoretic force is a possible explanation of this phenomenon. Gravity force and inertial force possibly act on the cells in the droplets as well as electrostatic force. These results suggest that the electric field is dominant for the behavior of cells in the droplet during the bouncing. It was previously unclear whether the electric field acts on the cells inside the droplet, but this finding indicates the existence of an electric field inside the droplet.

Fig 3 shows that instantaneous short-circuiting via droplets resulted in exogenous gene expression in Jurkat cells; however, gene transfection was not apparent after droplet bouncing. The essential difference between droplet bouncing and short-circuiting via a droplet is the presence or absence of a sufficient electrical current through the droplet. The cell suspension droplet was prepared with RPMI-1640 containing 0.1 M sodium chloride; therefore, the droplet can be considered as a conductive sphere. An electric circuit was always open during droplet bouncing, and then sufficient electric current could not flow. As shown in S2 Video, on the other hand, an electric current possibly flowed inside the droplet when short-circuiting occurs. Our previous report also showed that short-circuiting via a droplet showed little gene transfection when a solution with low electrical conductivity prepared with mannitol was used [35]. Furthermore, Joule heating in the droplets is also a possible factor for the electroporation. Tsong mentioned generating local Joule heating and inducing thermal phase transitions of the lipid bilayer by electric pulses [40]. The previous review also pointed out that various channels may be irreversibly denatured by Joule heating or electric modification of their functional groups. Therefore, the presence of a sufficient electrical current through the droplet could be required for gene electrotransfer.

As shown in Fig 3B, higher voltages resulted in significantly higher transfection efficiency, with a slight decrease in cell viability. In our previous investigation, which used human embryonic kidney (HEK) 293 cells and a different experimental apparatus, we showed that cell viability gradually decreased with increasing voltage applied, but significant differences in luciferase activities were not observed in a range of applied voltage of 2.5–3.5 kV. Increased voltages resulted in decreases in luciferase activity [35]. This discrepancy can be explained by differences in the principles of the assay. In the simultaneous evaluation of cell viability and exogenous gene expression in a 96-well assay system, the data obtained from a microplate reader are strongly affected by the recovery of the treated cells. As shown in S2 Video, short-circuiting induced droplet rupture. This phenomenon could affect the recovery of the cells. The density plots shown in Fig 3A indicate that most YFP-expressing cells were viable. Therefore, flow cytometric assays are more appropriate for the investigation of gene transfection, as presented in this paper.

Various electroporation methods have been previously developed and commercialized; cell viability and transfection efficiency are criteria for evaluating performance. The previous study demonstrated that the transfection efficiency of our method is comparable, as was cell viability for HEK293 cells [35]. In this study, we used Jurkat cells, and Figs 3 and 6 showed that the cell viability kept more than 75%, and the transfection efficiency was ranging from 8 to 35% 24 hours after the short-circuiting. Chicaybam et al. performed gene transfer into Jurkat cells by a commercial electroporation system (Lonza Nucleofector II), and the transfection efficiency was ca. 65%, but cell viability was less than 20% 24 hours after electroporation [41]. Although transfection efficiency depends on various experimental conditions such as the growth of the cells, and pulse parameters, our method has enough gene transfection performance for practical use.

Fig 4 shows transient membrane pore formation investigated by YO-PRO-1 uptake assays. We have already published the preliminary results [33, 35]; however, these results were also based on microwell-plate assays. Cell viability was not considered in these experiments. In this study, we performed YO-PRO-1 uptake assays using flow cytometry and 7-AAD. Fig 4 clearly shows that most YO-PRO-1 positive cells were viable, indicating that the increase in YO-PRO-1 fluorescence was due to the formation of transient membrane pores. The size of YO-PRO-1 (629.3 MW) is quite different from that of plasmid DNA (approximately 2–3 × 10^6^ MW). Therefore, we assumed that droplet bouncing could introduce small molecules into cells. As a result, Fig 4 shows that short-circuiting via a droplet stimulates YO-PRO-1 uptake; however, droplet bouncing did not increase in the population of YO-PRO-1 positive cells. Furthermore, we investigated the influx of calcium ions stimulated by droplet bouncing or short-circuiting. Ions are much smaller than YO-PRO-1 fluorescent dyes, so they can be used for detecting smaller pores. As a result, Fig 5 shows that short-circuiting stimulates the influx of calcium ions; however, droplet bouncing did not. Therefore, short-circuiting via aqueous droplets provoked transient membrane pores, but droplet bouncing did not deliver ions and YO-PRO-1 dyes. This result agreed with Fig 4E, showing that droplet bouncing did not result in successful gene transfection. Fig 6B also showed that droplet bouncing did not stimulate endocytosis. These results suggest that droplet bouncing did not deliver extracellular molecules via both transient membrane pore and endocytosis. In other words, our results lead to the conclusion that droplet bouncing does not deliver plasmid DNA into cells, although exogenous gene delivery was measured only using YFP expression. Gene electrotransfer investigated in most previous reports usually simultaneously stimulates transient membrane pore formation and exogenous gene expression. Therefore, it can be challenging to separate these phenomena. In addition, it has remained whether YO-PRO-1 uptake is stimulated by only transient membrane pore formation or not. The contribution of various channels and endocytosis to the transportation of small molecules into cells is not well studied. Further experiments need to be investigated to elucidate these points.

Fig 5A-2 showed that even short-circuiting via a droplet which did not contain calcium ions resulted in a slight increase in the population of Fluo-4 positive cells. The elevation of intracellular calcium ion concentration could be attributed to the release of Ca^2+^ from internal cellular stores to the cytoplasm [37].

Fig 6 suggests that the endocytotic pathway was stimulated by short-circuiting via an aqueous droplet. Endocytosis has recently emerged as a valuable molecular pathway for conventional gene electrotransfection; for example, pre-treatment of cells with inhibitors demonstrated the contribution of endocytosis [13, 17, 18]. This approach has also elucidated that cold atmospheric plasma irradiation stimulates endocytosis [42, 43]. Endocytosis is typically divided into four types: clathrin-mediated endocytosis, caveolae-mediated endocytosis, macropinocytosis, and phagocytosis [38]. M*β*CD extracts cholesterol from membranes and inhibits clathrin-mediated endocytosis, caveolae, and micropinocytosis. Fig 6A shows the effect on gene transfection of pre-treatment of cells with endocytosis inhibitors. As shown in Fig 6A-1, decreases in transfection efficiency suggest that cholesterol depletion is induced by M*β*CD, and these endocytotic pathways might be essential to gene electrotransfer. Pitstop 2-100 also produced significant decreases in transfection efficiency. Pitstop-induced inhibition of clathrin function interferes with clathrin-mediated endocytosis [39]. Fig 6A-2 indicated that clathrin-mediated endocytosis could be a possible molecular pathway for the exogenous gene expression investigated in this study.

In contrast to M*β*CD, Pitstop did not significantly decrease transfection efficiency by comparing 10 *μ*M and 20 *μ*M, shown in Fig 6A-2. This result suggests that inhibiting clathrin-mediated endocytosis did not completely block endocytosis; therefore, the other endocytotic pathways may have contributed to gene electrotransfer. The contribution of the other endocytotic pathways remains to be elucidated. Further experiments with different inhibitors for specific pathways will be investigated in the future.

Monitoring of endocytosis using pH-sensitive fluorescent dextran conjugates was also performed. Acidification of endocytic vesicles is one of the typical features of endocytosis. An increase in pHrodo Green fluorescence intensity indicates a pH decrease, so the encapsulation of macromolecules in endocytic vesicles can be monitored. Even if pHrodo Green dextran conjugates enter through the transient membrane pore, fluorescence intensity will not change. Therefore, it is difficult to conclude that pHrodo Green only enters via endocytosis; however, the pHrodo Green positive cells indicate the delivery of macromolecules by endocytosis. As shown in Fig 6B, short-circuiting resulted in significant increases in the population of pHrodo Green positive cells compared with the untreated control. This result also suggests that short-circuiting via an aqueous droplet stimulated endocytosis. Besides, we also performed the pHrodo Green experiments with M*β*CD. The results are shown in S1 Fig. The pre-treatment of Jurkat cells with M*β*CD resulted in an unexpected pHrodo Green fluorescence increase; however, an increase in the population of pHrodo Green positive cells by short-circuiting seems to be inhibited compared to the vehicle control. Further investigations are required to elucidate the effect of the endocytosis inhibitor on pHrodo Green uptake. The molecular weight of the pH-sensitive fluorescent dextran conjugates used in this study is around 10,000; therefore, extracellular molecules with greater than 10,000 MW, such as the dextran conjugate and plasmid DNA, could be delivered by the endocytotic pathway.

**Fig 7:**
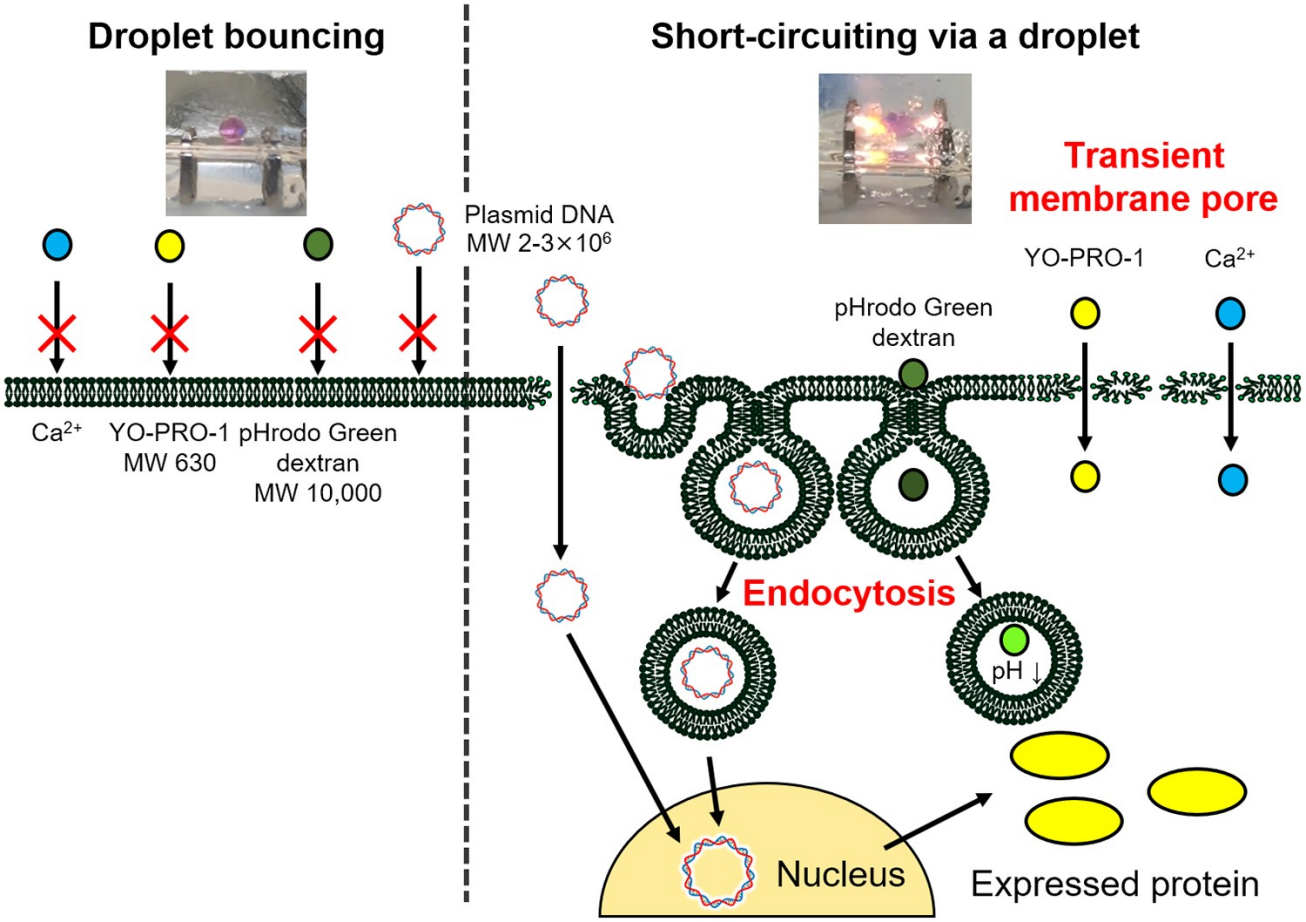
The possible mechanism for gene transfection of mammalian cells by short-circuiting via an aqueous droplet in dielectric oil. Short-circuiting stimulates transient membrane pore formation, shown by the influx of calcium ions and YO-PRO-1 uptake. Endocytosis, especially clathrin-mediated endocytosis, is essential in exogenous gene expression. Droplet bouncing does not deliver extracellular molecules.

The highlight of the present study are as follows: short-circuiting via an aqueous droplet containing Jurkat cells delivered calcium ions, YO-PRO-1, and plasmid DNA into the cells. Clathrin-mediated endocytosis, as well as the other pathways, contributed to exogenous gene expression. Droplet bouncing did not stimulate both transient membrane pore formation and endocytosis. These results are graphically summarized in Fig 7. However, the contribution of various channels and endocytosis to the transportation of small molecules into cells is needed to be studied.

## Conclusions

Flow cytometric assays were performed to elucidate the mechanism of gene delivery into mammalian cells by short-circuiting via water droplets in dielectric oil. Evaluation of cell viability and transfection efficiency showed that the type of HV applied strongly affected gene expression. Instantaneous short-circuiting via droplets resulted in exogenous gene expression in Jurkat cells; however, gene transfection was not apparent after droplet bouncing. YO-PRO-1 uptake and the influx of calcium ions demonstrated that short-circuiting induced transient membrane pore formation, but droplet bouncing did not. The short-circuiting via an aqueous droplet stimulated endocytosis. Endocytosis, especially clathrin-mediated endocytosis, may be essential in exogenous gene expression.

## Supporting information

S1 Video Typical droplet bouncing induced by applying DC HV electric field. A 3.0 *μ*l aliquot of RPMI-1640 was added to the silicone oil. 1.5 kV of DC HV was applied to the electrode with 5.08 mm gap.
(MP4)

S2 Video Typical short-circuiting via an aqueous droplet by applying DC HV electric field. A 3.0 *μ*l aliquot of RPMI-1640 was added to the silicone oil. 3.0 kV of DC HV was applied to the electrode with 5.08 mm gap. Following the short-circuiting, the DC HV power supply was turned off manually.
(MP4)

S3 Video Behavior of cells in a droplet around the anode at bouncing. A pair of electrodes with a pin-to-pin configuration (10 mm gap) was immersed in one well of a 12-well plate filled with silicone oil (20 cSt kinematic viscosity). A 3.0 *μ*l aliquot containing 3.0 × 10^4^ HEK293 cells in Opti-MEM (Thermo Fisher Scientific) was added to the silicone oil. 3.5 kV of DC HV was applied to the electrode to continue the droplet bouncing. The droplet bouncing was observed using an inverted microscope (IX60, Olympus) equipped with a 10×, 0.3 numerical aperture objective lens (UPlanFL, Olympus). The anode was located left side of the microscopic view. This movie was acquired a few minutes after starting the droplet bouncing by a high-speed camera (CHU30-C, Shodensha) at 30 frames per seconds (fps).
(MP4)

S4 Video Behavior of cells in a droplet around the cathode at bouncing. The droplet bouncing at the cathode was observed using an inverted microscope. The cathode was located left side of the microscopic view. This movie was acquired a few minutes after starting the droplet bouncing by the high-speed camera.
(MP4)

S1 Fig The effect of the pre-treatment of Jurkat cells with M*β*CD on monitoring endocytosis with pH-sensitive fluorescent dextran conjugates. Jurkat cells were pre-treated with 5 mM M*β*CD, before short-circuiting (3.0 kV, 5.08 mm gap, 1 short). Typical flow cytometry histograms are shown.
(TIF)
