# Supplementary figures and images for: Mechanistic studies of gene delivery into mammalian cells by electrical short-circuiting via an aqueous droplet in dielectric oil
Source: PLoS One. 2020 Dec 4;15(12):e0243361. doi: 10.1371/journal.pone.0243361 (PMC7717561; doi:10.1371/journal.pone.0243361)

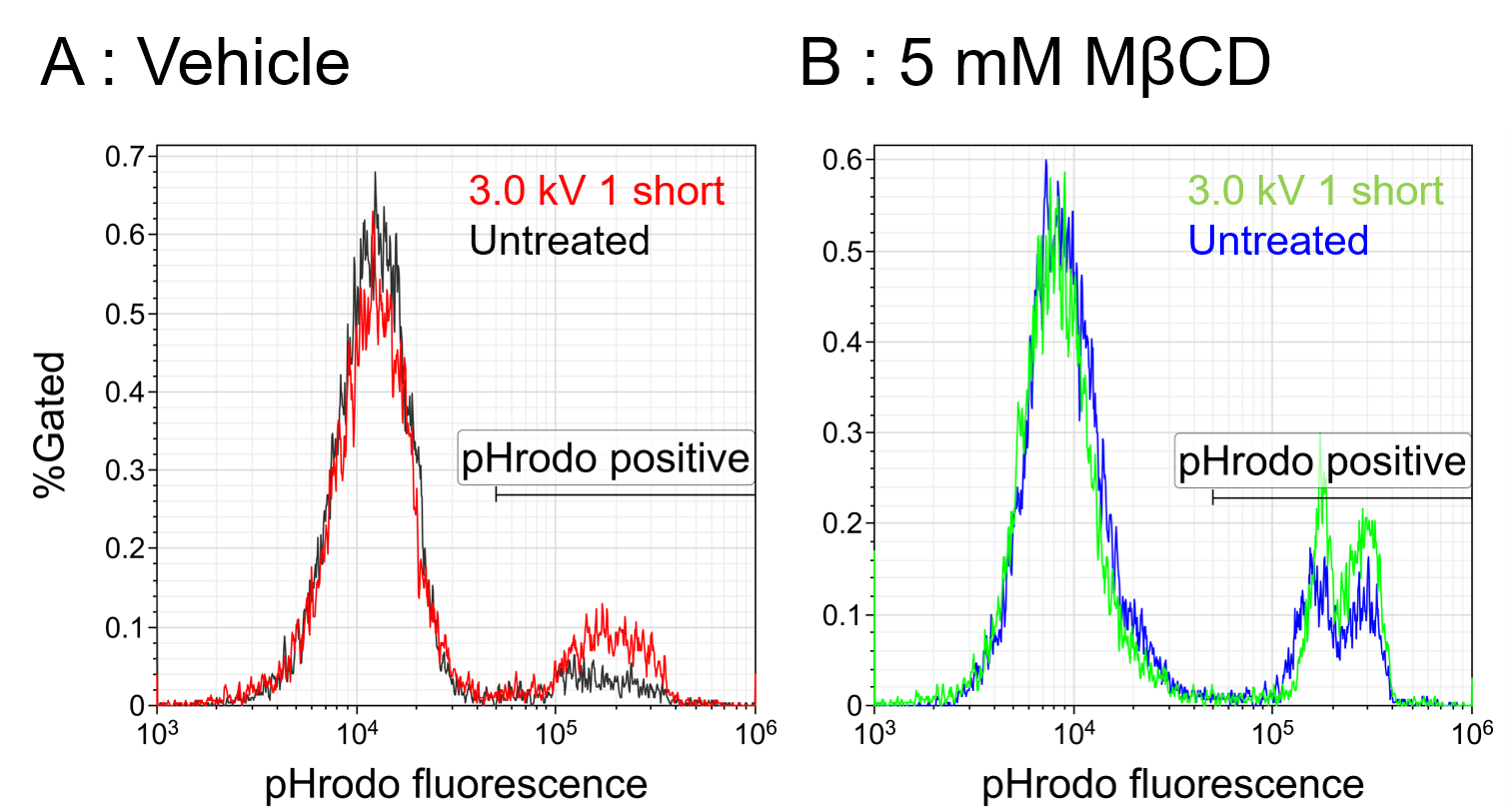

Supplement: S1 Fig — Jurkat cells were pre-treated with 5 mM MβCD, before short-circuiting (3.0 kV, 5.08 mm gap, 1 short). Typical flow cytometry histograms are shown. (TIF) [file pone.0243361.s005.tif]
